# Multi-modal representation learning in retinal imaging using self-supervised learning for enhanced clinical predictions

**DOI:** 10.1038/s41598-024-78515-y

**Published:** 2024-11-05

**Authors:** Emese Sükei, Elisabeth Rumetshofer, Niklas Schmidinger, Andreas Mayr, Ursula Schmidt-Erfurth, Günter Klambauer, Hrvoje Bogunović

**Affiliations:** 1 OPTIMA Lab, Department of of Ophthalmology and Optometry, Medical University of Vienna, Vienna, Austria; 2 LIT AI Lab, Institute for Machine Learning, Johannes Kepler University, Linz, Austria; 3 Institute of Artificial Intelligence, Center for Medical Data Science, Medical University of Vienna, Vienna, Austria

**Keywords:** Multi-modal imaging, Contrastive pre-training, Representation learning, Predictive modeling, Retinal imaging, Medical imaging, Translational research, Retinal diseases, Machine learning

## Abstract

Self-supervised learning has become the cornerstone of building generalizable and transferable artificial intelligence systems in medical imaging. In particular, contrastive representation learning techniques trained on large multi-modal datasets have demonstrated impressive capabilities of producing highly transferable representations for different downstream tasks. In ophthalmology, large multi-modal datasets are abundantly available and conveniently accessible as modern retinal imaging scanners acquire both 2D fundus images and 3D optical coherence tomography (OCT) scans to assess the eye. In this context, we introduce a novel multi-modal contrastive learning-based pipeline to facilitate learning joint representations for the two retinal imaging modalities. After self-supervised pre-training on 153,306 scan pairs, we show that such a pre-training framework can provide both a retrieval system and encoders that produce comprehensive OCT and fundus image representations that generalize well for various downstream tasks on three independent external datasets, explicitly focusing on clinically pertinent prediction tasks. In addition, we show that interchanging OCT with lower-cost fundus imaging can preserve the predictive power of the trained models.

## Introduction

Deep learning techniques have significantly advanced in various medical image interpretation tasks^1–4^. However, building robust and generalizable deep-learning models often demands a substantial volume of labeled data. For instance, in their seminal work, Esteva et al.^1^ compiled a dermatologist-labeled dataset of 129,450 clinical images to reach on-par performance with experts in classifying skin cancer. Compiling large datasets with diverse cases is a laborious and time-consuming task, and it can also introduce annotator biases. As such, the progress of supervised deep learning models for medical imaging is hindered by the limited availability of costly, extensively labeled datasets^5^. Self-supervised learning (SSL) approaches aim to overcome these challenges by learning meaningful representations from large amounts of unlabeled data, reducing the reliance on costly and time-consuming expert annotations while improving the accuracy and generalizability of predictive tasks^6^.

The fundamental concept behind SSL is to create auxiliary or pretext tasks that enable the model to learn meaningful and valuable representations directly from the data without relying on human annotations. Once the model acquires these representations through pretext tasks, the learned features can be transferred to downstream tasks, such as classification, segmentation, or detection, where labeled data is often scarce. These approaches usually utilize discriminative modeling, reconstruction tasks, or contrastive learning techniques^7–10^, enabling efficient downstream task learning with fewer labeled examples. These methods have demonstrated substantial performance improvements not only in the natural image domain but also in medical imaging, enhancing both classification^11,12^ and segmentation^13–15^ tasks. Nonetheless, they often require large datasets for effective pre-training, which can be challenging in medical domains where privacy concerns restrict data collection and sharing. Additionally, many existing SSL models focus on single-modality inputs, limiting their ability to fully capture the complementary information available from multiple modalities.

In the medical workflow, clinicians regularly interpret a combination of multiple modalities to deliver comprehensive patient care, such as clinical notes, laboratory tests, vital signs, medical images, genomics, and more. However, conventional machine learning applications often concentrate on single modalities, leading to inflexible models that struggle to adapt to other tasks or varying data distributions for the same task without retraining. The recent emergence of *foundation models* exploits the multi-modal nature of available medical data and has gained considerable attention^16–18^. These models, by definition, pre-trained encoders adaptable or fine-tunable for many tasks, show great promise^19^. They are of particular interest for their effectiveness in deciphering intricate structures within multi-modal data, making them well-suited to successfully address a broad range of challenging tasks in the medical domain^20–23^.

Ophthalmology is an image-intensive sub-specialty at the forefront of integrating artificial intelligence (AI) into medical practice^24–27^. The eye and its intricate retina offer a unique advantage, directly observable through non-invasive imaging methods such as color fundus photography and, more recently, optical coherence tomography (OCT). The 3D OCT provides micrometer-level resolution within a volumetric scan, achieving widespread adoption and emerging as the gold standard for managing retinal diseases. Combining 2D color fundus photography or near-infrared reflectance imaging with 3D OCT confers a holistic insight into the retinal structure^28^; hence, every OCT scanner on the market takes a fundus image in addition to the OCT scan as part of the acquisition. However, expert annotations are not always readily available for these imaging modalities, which can hinder the training of supervised AI models in clinical practice. The automatic availability of these two modalities, OCT and fundus images, nonetheless, offers an opportunity for multi-modal SSL methods. Learning meaningful representations by jointly modeling the different imaging modalities can, in turn, facilitate disease progression modeling and personalized patient management. Despite this potential, the realm of multi-modal SSL in ophthalmology remains relatively uncharted, as current methodologies primarily focus on fusion techniques^29,30^ or uni-modal setups^31–34^.

Motivated by this, our research focuses explicitly on the multi-modal advantages of pairing OCT volumetric data with fundus images. To this end, we base our analysis on multi-modal SSL techniques like Contrastive Language-Image Pre-training (CLIP)^35^ and Contrastive Leave One Out Boost (CLOOB)^36^, that have proven their efficacy in acquiring transferable representations when trained on both textual and visual data and are expected to hold immense promise in medical imaging. So, unlike approaches that focus solely on 2D information or neglect the complementary insights provided by aligning fundus images with OCT scans of the same eye, our methodology seeks to leverage the synergies between these modalities to capture complex patterns and representations applying these paradigms to retinal imaging in a multi-modal fashion. By doing so, we anticipate generating more meaningful and transferable representations of the retina, ultimately enhancing the predictive models’ overall performance.

Our contributions can be summarized as follows:

1. We introduce a novel multi-modal contrastive pre-training method for retinal imaging based on the CLIP and CLOOB paradigms, combining the 2D and 3D information available from fundus and OCT imaging.
2. We extensively evaluate the pre-trained encoders’ performance on three independent external datasets at adapting to a series of clinically relevant prediction tasks: i) *regression*: structure-function and biomarker measurements, ii) *classification*: biomarker detection, and disease diagnosis, and iii) *forecasting*: disease and treatment prognosis.
3. We compare our proposed approach with fully supervised training, natural image-based pre-training, and uni-modal SSL pre-training, offering insights into its relative effectiveness and demonstrating its potential advantages in enhancing predictive models for ophthalmic applications.
4. We demonstrate how the *multi-modal contrastive pre-training* enhances the predictive performance of 2D fundus image-based models. Furthermore, we show how it allows, to a certain extent, the interchanging of the two imaging modalities when the encoder weights are frozen for supervised predictive training. Fundus image-based predictions are of strong interest as fundus imaging tools are more cost-effective and widely accessible for diagnostics in ophthalmology than OCT scanners.

## Materials and methods

### Datasets

We used different datasets for the contrastive pre-training and the supervised downstream tasks. Here, we list the datasets and the trial numbers, while a detailed overview of the dataset properties from each study and the cohorts’ demographic and clinical data at the time of baseline OCT acquisition is provided in Supplementary Table A1. Ethics approval for post-hoc analysis of the datasets was obtained from the Ethics Committee at the Medical University of Vienna (MUW), Austria (EK: 1246/2016). This work adhered to the tenets of the Declaration of Helsinki and the MUW’s standards of good scientific practice.

For *pre-training*, we used large-scale longitudinal data from the imaging data collection of clinical studies available at OPTIMA Lab, MUW. We extracted 153,306 fundus photography/near-infrared reflectance imaging scans and the corresponding OCT volumes from 3,790 patients diagnosed with neovascular age-related macular degeneration (AMD). The scans were acquired using Spectralis, Cirrus, Nidek, or Topcon scanners at different sites. The majority of the imaging data were prospectively collected during five randomized multi-center clinical trials: FLUID (NCT01972789), TREND (NCT01948830), OCTAVE (NCT01780935), HAWK (NCT02307682) and HARRIER (NCT02434328).

For fine-tuning and validation of the proposed supervised models on *clinical downstream tasks*, we used the retrospective de-identified HARBOR clinical trial dataset (NCT00891735)^37^ (51,186 scans from 2,183 unique eyes of 1,094 patients with AMD), the publicly available OLIVES dataset^38^ (1,590 scans from 96 unique eyes of 87 patients with diabetic retinopathy (DR)), and a mixed diseases dataset from the OPTIMA Lab imaging datasets (1,922 scans from 1,922 unique eyes of 1,922 patients), which is referred to as MIX in the rest of the paper. The latter consisted of a selection of baseline scans from clinical studies covering 4 different retinal conditions: diabetic macular edema (DME), intermediate age-related macular degeneration (iAMD), retinal vein occlusion (RVO), geographic atrophy (GA), and healthy cases. Hence, our assessment of transferability was based on various datasets extending to diverse clinical settings and disease types.

Among the different datasets, the scanning protocols varied, leading to different OCT volume resolutions with 21-256 B-scans, with each B-scan having a size in the range of 256-1,024$\times$480-1,024 pixels with a pixel size of 2.13–23.43 $\times$ 1.95–4.19 $\upmu$m$^2$. Similarly, the size of the fundus images was in the range of 290–2,000 $\times$ 348–2,992 pixels.

#### Data processing and augmentation

We re-scaled the fundus images and the OCT B-scans to 224×224 to allow larger batch sizes, which is especially important for contrastive pre-training. For OCT volumes, we then uniformly sample 20 B-scans - preserving the order of B-scans - to account for the variability in the number of B-scans used during image acquisition. This can lead to potentially missing variations and abnormalities in other parts of the volume; however, it still captures information from different locations within the volume, which helps in understanding the 3D morphology of the tissue, which is critical for many clinical and research applications. The B-scans and near-infrared reflectance fundus images are inherently grayscale; however, the color fundus photographs from the Topcon scanners were converted to grayscale. Finally, the images/volumes were normalized by intensity mean and variance, image/volume-wise.

Additionally, random image transformations are used for the uni-modal contrastive pre-training to create different views of the same image/volume. For this, we followed the SimCLR^39^ framework without the transformations that do not apply to the data at hand, resulting in (1) centered crop, (2) horizontal flip, (3) Gaussian blur, and (4) contrast adjustment. The first two transformations were applied with a probability of 0.5, while the Gaussian blur and contrast adjustment with a probability of 0.3. Finally, the images/volumes were normalized, image/volume-wise.

#### Data partitioning and stratification for experimental setup

We partitioned the respective datasets into non-overlapping training-validation-test partitions for each experimental setup at a ratio of 80%–15%–5% for the *contrastive pre-training task* and 80%–10%–10% for *the external downstream tasks* (Supplementary Figure A1). We created a patient-level separation while dividing the data into subsets, ensuring a stringent evaluation of the models. As the labeled datasets were generally imbalanced, we split them using a stratification technique for the downstream tasks, ensuring the same proportion of target labels in each subset.

**Figure 1:**
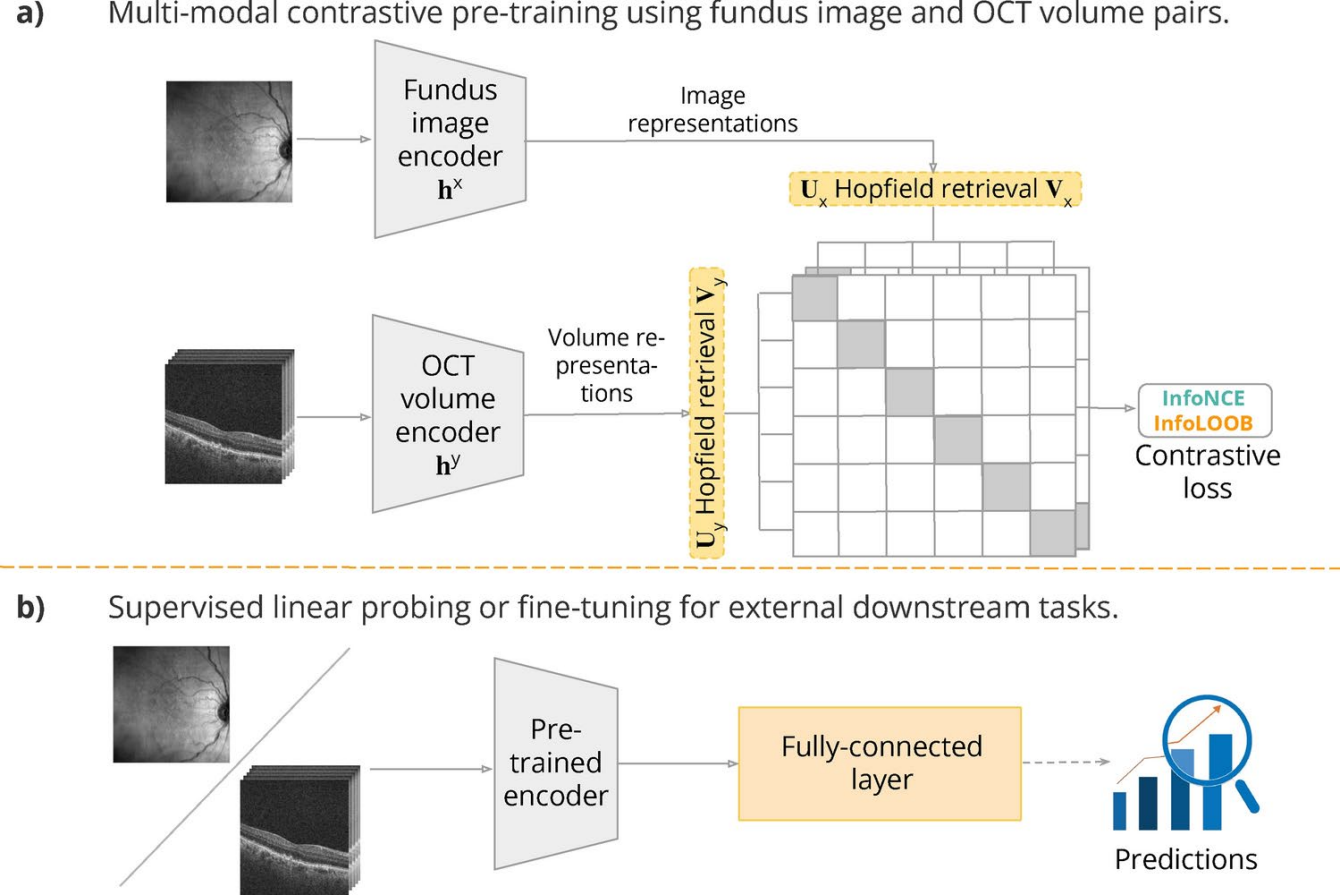
The proposed CLIP/CLOOB framework: contrastive pre-training of the encoders ($h^{\textbf{x}}, h^{\textbf{y}}$) of the two retinal imaging modalities (fundus images—$\textbf{x}$ and OCT volumes—$\textbf{y}$), followed by using the pre-trained encoders for downstream predictive tasks.

### Methodology

As shown in Fig. 1, our proposed framework consists of pre-training and downstream phases. In the first phase, the model learns a transferable fundus image/OCT volume representation via contrastive learning. In the downstream phase, clinically relevant prediction tasks are conducted by either linear probing or fine-tuning the learned encoder with often limited labeled data. Beyond disease classification, we aim to explore a wider range of clinically relevant prediction tasks, such as structure-function and biomarker measurements or disease evolution forecasting, broadening the potential applications of these enhanced models.

#### Contrastive pre-training

Contrastive learning is a learning paradigm that extracts rich and transferable representations. The central idea of contrastive learning is that matched data points should yield similar representations, so-called embeddings, while unmatched data points should have a low similarity. In multi-modal contrastive learning, CLIP has emerged as a widely adopted and effective approach. Its fundamental goal is to concurrently train modality-specific encoders, aligning each modality to a shared embedding space through the InfoNCE contrastive objective^40–42^. This objective is designed to bring representations of distinct modalities within matched data points, such as text describing a specific image, into close proximity in the embedding space. Conversely, representations of unmatched data points, like an image and text describing an entirely different one, should be positioned considerably far from each other.

While CLIP has been highly successful and widely used, it has been shown to suffer from the *explaining away* problem^36^, in which a few features are overemphasized while others are neglected. This occurs when learning focuses on only a subset of features or when the covariance structure in the data is insufficiently extracted, often due to the saturation of the InfoNCE learning objective. Fürst et al.^36^ introduced CLOOB to overcome this problem. It employs a *modern Hopfield network*^43^ to enhance the capturing of covariance structure during learning. Additionally, CLOOB employs the InfoLOOB^41^ objective to avoid saturation issues associated with the InfoNCE objective used in CLIP.

Our proposed contrastive framework, depicted in Fig. 1**a**, utilizes the CLIP and CLOOB pre-training techniques. During pre-training, the aim is to bring the embeddings of paired fundus and OCT scans of the same eye, acquired at the same visit close while pushing the negatives, hence unpaired fundus and OCT scan embeddings further apart. This allows for learning representations for both modalities explicitly containing information about both.

We employ ResNet18 with pre-trained ImageNet weights as the backbone image encoder and VideoResNet18 with pre-trained Kinetics^44^ weights as the backbone volume encoder for fundus images and OCT volumes, respectively. We start from pre-trained weights as they provide a good starting point for training, significantly speeding up the convergence of the model. The dimension of the embedding space was set to $d = 512$, which determines the output size of both encoders. The hyper-parameters and training strategies suggested by OpenCLIP^45^ and CLOOB were used (Supplementary Table A2).

The models were trained with mixed precision for 300 epochs in a distributed fashion on three NVIDIA A100 80GB GPUs with batch size 128 on each GPU. To speed up the training and decrease the memory requirements, a single randomly selected fundus image - OCT volume pair per patient was shown to the model at each epoch, with the random seed being adjusted according to the current time. The model weights at the final epoch were saved as the checkpoint for adapting to downstream tasks. As primary evaluation metrics during the contrastive pre-training, we tracked the evolution of InfoNCE and InfoLOOB losses. Additionally, we analyzed the distribution of cosine similarities of the embedded fundus and OCT imaging modalities. Furthermore, we computed the top-*k* retrieval accuracy metric to evaluate the ability of the model to retrieve the corresponding fundus image - OCT volume pairs.

#### Adaptation to downstream tasks

After contrastive pre-training, the encoders were used to extract lower-dimensional feature representations for downstream predictive tasks. We added a single fully connected layer after the encoder block (Fig. 1b) as our prediction head. To demonstrate the models’ feature extractor capabilities, *linear probing* was performed by freezing the encoder weights and training only the last layer. Additionally, we conducted experiments by *fine-tuning* the entire model for the downstream tasks.

We have outlined a range of clinically relevant downstream tasks on three external datasets not used for pre-training, including disease diagnosis, biomarker detection, disease and treatment prognosis, and structure-function prediction. These tasks cover *regression, classification*, and *forecasting* problems, as described below:

- **Regression tasks: structure-function and biomarker measurements. **The HARBOR and OLIVES datasets include information on best-corrected visual acuity (BCVA) and central subfield thickness (CST). BCVA quantifies overall visual function, reflecting the sharpness of vision with optimal correction, often via glasses or contact lenses, and is assessed using a Snellen chart. CST, obtained via OCT imaging, gauges the thickness of the central subfield in the macula, which serves to diagnose conditions like diabetic macular edema. These measurements are pivotal in diagnosing, treating, and monitoring eye conditions. They assist ophthalmologists in evaluating visual function, detecting anomalies, and planning appropriate interventions, thus enhancing clinical workflows. Here, we aim to provide them with an automated assessment tool.
- **Classification tasks: biomarker detection and disease diagnosis.** We formulated an image classification task on the HARBOR dataset to detect fluid accumulation within choroidal neovascularization (CNV) (Fluid present). This task involves identifying the presence of fluid in OCT, as determined by a certified reading center, which may manifest as subretinal fluid, intraretinal fluid, or cystoid macular edema. Although this is an OCT-related biomarker, we hypothesize that the multi-modal pre-training could also allow for predicting it accurately from fundus images, offering a cost-effective screening tool in practices where OCT scanners are unavailable.
  In the context of the OLIVES and MIX datasets, we focus on the task of disease classification. In the former, we want to identify the presence of DME or DR, while the MIX dataset allows for defining a multi-class problem. This entailed distinguishing between healthy cases and those involving DME, iAMD, RVO, and GA. Automated disease diagnosis from retinal imaging is essential in ophthalmology as it enables accurate and timely identification of various conditions, facilitating early intervention and personalized treatment strategies for improved patient outcomes.
- **Forecasting tasks: disease and treatment prognosis. **In the HARBOR dataset, we utilized the effective number of injections ($n_{inj}$) received during the 2-year study period under the pro-re-nata treatment regimen to establish distinct treatment requirement categories, much akin to the approach taken by Romo *et al.*^46^. Specifically, we categorized patients into *high* ($n_{inj} \ge 16$) and *low* ($n_{inj} \le 5$) treatment requirement groups, where the high category encompassed patients in the first quartile of the population, and the low category included those in the third quartile. Here, we focus on predicting the *high* treatment need (High TN) category for our forecasting task after the administration of loading doses. Establishing treatment requirement categories, such as high and low treatment needs, can be crucial in optimizing patient care and treatment planning, ensuring timely and personalized interventions.
  Furthermore, within this dataset, the fellow eyes of a subgroup of patients exhibited a conversion to geographic atrophy (GA Conv.) or choroidal neovascularization (CNV Conv.) within the two-year duration of the trial. Consequently, we introduced an additional downstream forecasting task. Specifically, we framed the problem as predicting the conversion to GA/CNV based on the baseline scan data of these fellow eyes. Predicting the conversion to GA or CNV in fellow eyes provides valuable insights for proactive management and early detection of progressive retinal conditions, contributing to improved patient outcomes.

The training was run on an NVIDIA A100 40GB GPU with a batch size of 128 (fundus input)/64 (OCT input) in the linear-probing setup and 64 (fundus input)/32 (OCT input) in the fine-tuning setup, while the learning rate was found by performing a hyper-parameter search on the $[1e-6, 1e-3]$ interval. We adopt an AdamW optimizer^47^ with the initial learning rate found during the hyper-parameter search and a weight decay of 0.1. Moreover, a reduce-on-plateau learning rate scheduler was applied. The training was performed for a maximum of 100 epochs or until the training loss demonstrated convergence on the validation data. Early stopping was applied to avoid overfitting: the training was stopped if the loss on the validation set no longer decreased for five epochs. We used mean squared error loss for the regression tasks, while binary cross-entropy and cross-entropy losses in the classification and forecasting tasks, respectively.

The $R^2$-score and the root-mean-square error (RMSE) were used to evaluate the regression models. In contrast, the weighted area under the receiver operating characteristic curve (AUROC) and average precision score (AP) were used to assess the performance of the models for classification. We computed the weighted one-vs-rest AUROC and AP scores for the multi-class setup. We applied bootstrapping on the hold-out test sets to estimate the models’ performance variance.

#### Uni-modal baselines

Our multi-modal pre-training was benchmarked against three pertinent uni-modal SSL models-SimCLR^39^, BYOL^48^, and VICReg^49^. SimCLR focuses on maximizing agreement between augmented views of the same image, learning representations by contrasting positive pairs against a large set of negative pairs. BYOL employs a dual neural network architecture, where one network predicts the other’s representations, fostering self-supervision without the need for negative pairs. VICReg introduces a variant of contrastive learning with virtual instances, focusing on leveraging only positive pairs in its training process by incorporating virtual augmentations. Its specially designed objective function serves to prevent dimension and mode collapse, addressing challenges associated with other contrastive learning methods and ensuring the stability and efficacy of the learning process. Comparing these methods allows for an assessment of the effectiveness of our CLIP/CLOOB-based approach in the context of retinal imaging and understanding whether combining 2D and 3D information provides advantages over more traditional uni-modal SSL techniques.

For all uni-modal methods, we utilized ResNet18 and VideoResNet18 as encoders. While the multi-modal pipeline does not rely on an additional projection layer, the uni-modal approaches do. For this, we used a single 128-dimensional linear layer that was then discarded for the downstream tasks. These SSL models were trained on the same pre-training dataset using identical hyper-parameters and optimizer setup as our multi-modal method, except for the batch size, which had to be adjusted according to the computational resource limitations. In the 2D setup, the batch size could be increased to 256 per GPU, while in the 3D setup, it had to be decreased to 80.

#### Interchanging retinal imaging modalities

We analyzed the feasibility of seamlessly swapping OCT embeddings with fundus embeddings for the OCT-based trained prediction models. This approach explores whether models designed for one imaging modality can generalize across different modalities without requiring extensive re-training, which could simplify model deployment in clinical settings. To test this hypothesis, we extracted the latent embeddings of the fundus images within the test sets of the respective downstream tasks using the frozen pre-trained fundus encoder. Subsequently, we passed these fundus embeddings as input to our OCT-based prediction models, which had been trained using the linear probing setup. Linear probing preserves the contrastive pre-training constraints, precisely the objective of mapping corresponding fundus image and OCT volume pairs closer together. As such, it serves as an ideal testing ground for evaluating our hypothesis of imaging modality interchangeability. The ability to seamlessly utilize embeddings from one modality as input for models trained on a different modality implies greater flexibility in clinical applications, potentially reducing the need for modality-specific models and enabling a more streamlined and versatile approach to predictive tasks in retinal imaging.

## Results

### Multi-modal contrastive pre-training yields an effective retrieval system

First, we evaluate the effectiveness of the pre-training by assessing the model’s proficiency in performing the pre-training task, namely, retrieving the corresponding OCT volume for a given fundus image from the same eye and vice versa using the top-*k* accuracy score. Top-1 accuracy refers to the accuracy rate that the class with the highest probability is the correct class, while for $k > 1$, top-*k* accuracy refers to the accuracy rate that the top-*k* pairs with the highest probability contain the correct pair. To intensify the challenge, we enhance the task complexity by considering all samples per patient. It is important to note that this task is nearly impossible for human experts to perform accurately.

**Table 1:** Results of the retrieval task. Given a fundus image, the correct OCT volume must be selected from a set of candidates. Top-1, top-5, and top-10 accuracy are shown for the hold-out test set, along with the upper and lower limits for a 95% confidence interval (CI). Here, Baseline refers to the encoders initialized with ImageNet/Kinetics-pre-trained weights, CLIP and CLOOB to the encoders obtained using the CLIP- and CLOOB-based pre-training, respectively. The best performance for each setup is indicated in bold.

Retrieval task	Test set	Model	**Accuracy score**		
			Top-1	Top-5	Top-10
Fundus to OCT	One sample/patient	Baseline	0.005 (0.000, 0.040)	0.022 (0.005, 0.062)	0.041 (0.016, 0.092)
		CLIP	0.781 (0.704, 0.848)	**0.959 (0.908, 0.984)**	**0.980 (0.938, 0.995)**
		CLOOB	**0.800 (0.720, 0.861)**	0.947 (0.898, 0.979)	0.974 (0.927, 0.992)
	All samples/patient	Baseline	0.000 (0.000, 0.001)	0.001 (0.000, 0.002)	0.002 (0.001, 0.003)
		CLIP	0.097 (0.090, 0.104)	0.310 (0.299, 0.321)	0.457 (0.445, 0.468)
		CLOOB	**0.109 (0.102, 0.117)**	**0.333 (0.322, 0.344)**	**0.482 (0.470, 0.494)**
OCT to fundus	One sample/patient	Baseline	0.005 (0.000, 0.040)	0.025 (0.005, 0.062)	0.050 (0.021, 0.102)
		CLIP	0.768 (0.689, 0.836)	**0.949 (0.898, 0.979)**	**0.978 (0.938, 0.995)**
		CLOOB	**0.799 (0.720, 0.861)**	0.945 (0.889, 0.975)	0.973 (0.927, 0.992)
	All samples/patient	Baseline	0.000 (0.000, 0.001)	0.001 (0.000, 0.001)	0.001 (0.001, 0.003)
		CLIP	0.093 (0.086, 0.100)	0.293 (0.283, 0.304)	0.437 (0.425, 0.448)
		CLOOB	**0.103 (0.096, 0.110)**	**0.334 (0.323, 0.345)**	**0.484 (0.473, 0.496)**

**Figure 2:**
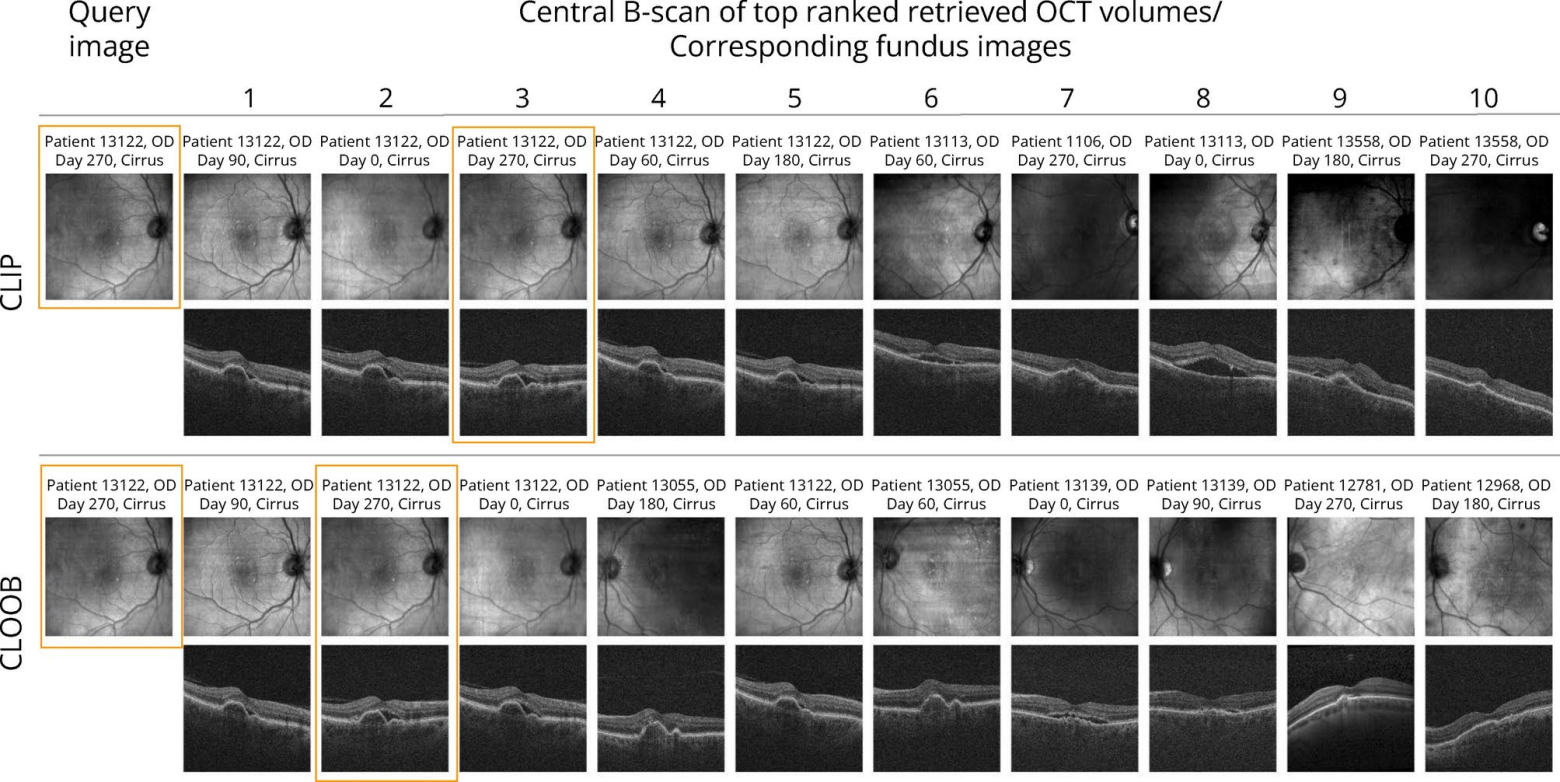
Example results for the retrieval task on a hold-out test set. The 10 OCT volumes for which representations are the most similar to the query fundus image are shown along with their corresponding fundus images. Orange bounding boxes mark the correct pair.

The top-*k* accuracy scores for the contrastive models on the retrieval task, with *k* values of 1, 5, and 10, are summarized in Table 1, and illustrative examples are presented in Fig. 2. A retrieval score of less than 0.1% in the baseline setup (ImageNet/Kinetics initialized encoders) proves that the corresponding image-volume pairs are not consistently mapped close in the latent space by default, which is what we expect. In the more straightforward, single sample per patient case (189 fundus image - OCT volume pairs from 189 patients), CLIP ranked the correct OCT volume first in 78.1% of cases, while CLOOB ranked the correct OCT volume first in 80% of cases, the top-5 and top-10 accuracy being above 94% for both. The models’ ability to precisely retrieve the corresponding OCT volume from a given fundus image, and vice versa, establishes a significant opportunity for interchangeability between OCT and fundus embeddings within the downstream prediction tasks.

On the complete hold-out set of 6,948 fundus image and OCT volume pairs, CLIP ranked the correct OCT volume first in 9.70% of cases, while CLOOB ranked the correct OCT volume first in 10.90% of cases. Moreover, we found that, on average, 58.20% of the retrieved images/volumes came from the same patient in the case of CLIP and 62.90% in the case of CLOOB, suggesting that patient scans from different time points are inherently mapped closely together in both models. However, preliminary cluster analysis of these representations revealed no interpretable insights. Nonetheless, it opens opportunities for further investigation.

With the inherent characteristics of contrastive pre-training, we expected that the embeddings for the two distinct imaging modalities would interweave, leading to larger cosine similarities between the positive pairs. The embeddings derived from CLIP and CLOOB had similarity scores of 0.698 (SD = 0.084) and 0.385 (SD = 0.072), respectively, while for the embeddings obtained with the ImageNet/Kinetics pre-trained encoders were only 0.108 (SD = 0.000). This behavior indicates that scans from the same eye (positive pairs) cluster closely together, while those from different eyes (negative pairs) disperse more widely (Supplementary Fig. A2a). Moreover, we have empirically observed that the model primarily segregates the scans based on the vendor (Supplementary Fig. A2b). This outcome was somewhat expected since scans obtained from different scanners exhibit significant dissimilarities, and accounting for them was out of the scope of this work.

### The learned representations improve predictive performance on external downstream tasks

**Figure 3:**
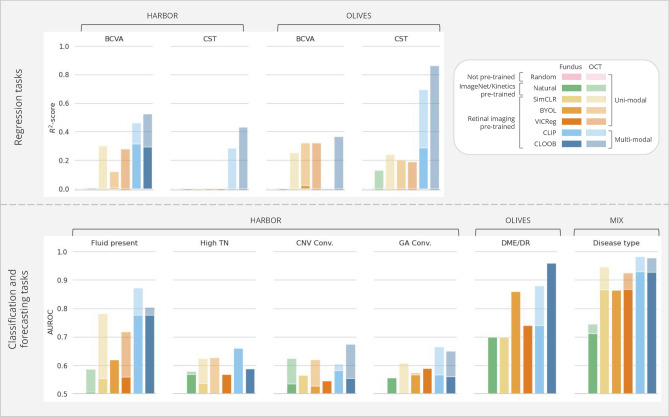
The predictive models’ average performance on the external downstream tasks in *linear probing*, evaluated on the hold-out test set via bootstrapping-based validation. Fundus image-based results are depicted as overlapped over the OCT volume-based results, colored less opaquely. Supplementary Table A3 provides a detailed overview of the numerical results.

The outcomes of the linear probing experiments illustrate the advantages of domain-specific self-supervised pre-training over natural image-based or uni-modal contrastive pre-training for the encoders across most downstream tasks in both the *regression* and *classification/forecasting* configurations (Fig. 3). As anticipated, the models based on the more detailed OCT scans exhibited superior performance compared to their fundus imaging-based counterparts in all tasks, albeit with a minimal margin in some classification instances. This suggests that the learned fundus embeddings benefit from the richer information in the higher-dimensional OCT volumes.

In tasks involving *regression* based on fundus images, where many models struggle to capture the variance in the dependent variable, resulting in negative $R^2$-scores indicative of predictive power worse than a simple average, the adoption of multi-modal pre-training proves to be an effective strategy. Specifically, in predicting BCVA from fundus images in the HARBOR dataset, only models pre-trained with CLIP and CLOOB yielded positive $R^2$-scores. The CLOOB pre-trained model demonstrated similar magnitudes when using OCT volumes as inputs. Notably, the CLIP-based model achieved the highest $R^2$-scores of 0.308 for fundus images and 0.462 for OCT volumes. Similarly, in CST prediction, effective explanatory power for the dependent variable was primarily achieved using OCT volumes and multi-modal pre-trained weights. On BCVA prediction tasks with fundus inputs in the OLIVES dataset, multi-modal approaches were outperformed by BYOL and VICReg pre-trained models, with only BYOL achieving a positive albeit low $R^2$-score. CLIP-based pre-training was best for predicting CST from fundus images, reaching an $R^2$-score of 0.286. In contrast, using OCT volumes as inputs significantly improved performance, with CLOOB-based models achieving the highest scores of 0.364 and 0.864 for BCVA and CST, respectively.

Across all downstream *classification* and *forecasting* tasks, models leveraging multi-modal pre-training consistently demonstrated superior performance compared to their uni-modal counterparts, albeit by a marginal increase in certain instances. Notably, the ability to predict the presence of fluid from fundus images using the multi-modal pre-trained encoders approached the efficacy of OCT-based methods. The best performance was achieved by the CLIP-based pre-training, leading to an AUROC of 0.755, while in the OCT-based case, CLOOB yielded an AUROC of 0.871. This development holds promise for an essential pre-screening tool based on fundus images, a modality that, as previously discussed, is more cost-effective and widely accessible.

While the models exhibited diminished performance in more intricate *forecasting* tasks, stratifying treatment requirements proved more manageable, particularly with OCT volumes. The CLOOB-based model achieved an AUROC of 0.755, outperforming other tasks. Predicting whether an eye will converge to GA or CNV within 24 months poses an ambitious task due to the many changes in the diseased retina over such an extended time frame. Nevertheless, the multi-modal pre-trained models showcased their superiority even in these challenging tasks. Interestingly, fundus-based models initialized with uni-modal pre-trained weights slightly outperformed their OCT-based counterparts in specific scenarios. For instance, in the case of CNV convergence, SimCLR using fundus images as inputs achieved an AUROC score of 0.565, surpassing the OCT-based model, which attained only 0.513. This suggests that the learned 3D representations may not fully encapsulate patterns relevant to this task.

**Figure 4:**
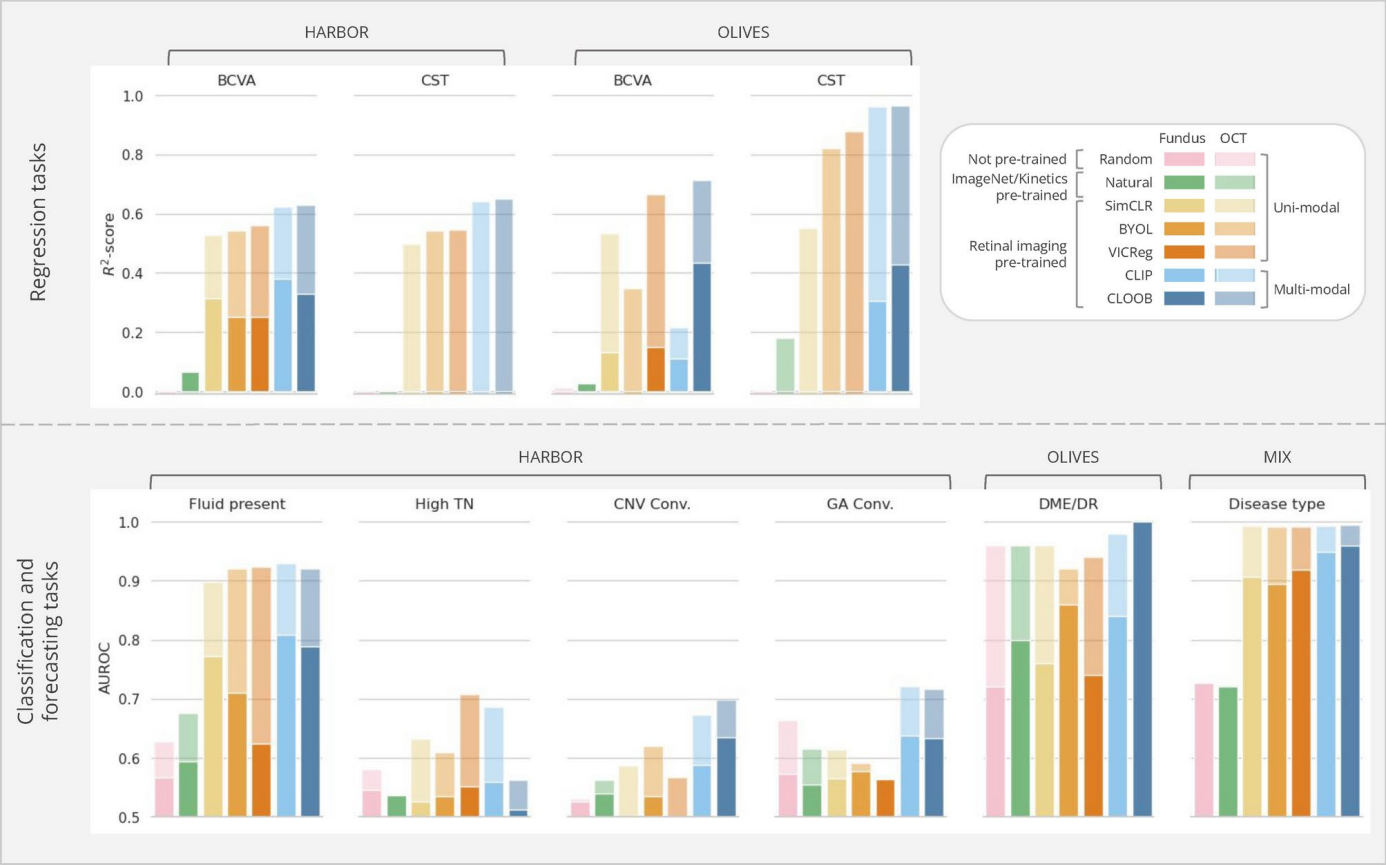
The predictive models’ average performance on the external downstream tasks in *fine-tuning*, evaluated on the hold-out test set via bootstrapping-based validation. Fundus image-based results are depicted as overlapped over the OCT volume-based results, colored less opaquely. Supplementary Table A4 provides a detailed overview of the numerical results.

The disease classification tasks have the highest performance on both the OLIVES and MIX datasets. Despite not being exposed to non-AMD cases during pre-training, the resulting latent representations of fundus images and OCT scans depicting various diseases within external datasets demonstrate clear separability (Supplementary Fig. A2). This implies that the latent representations effectively capture disease-related patterns and generalize well to domain shifts. Furthermore, achieving an AUROC above 0.900 with fundus-based classifiers again provides a good basis for adopting such models in clinical practices, particularly in scenarios where the more expensive OCT modality is not readily available.

Comparing the linear probing results with the outcomes from the fine-tuning setup reveals that the linear probing performances using multi-modal pre-trained encoders closely approach those achieved through fine-tuning (Fig. 4). Hence, these pre-trained models could readily be used as feature extractors even without needing strong computational resources to fine-tune the whole model. In the case of the *classification* and *forecasting tasks*, the CLIP or CLOOB pre-trained models always outperform the uni-modal and fully-supervised approaches. However, the results of the regression tasks in the HARBOR dataset for the BCVA task resulted in notably better performance with SimCLR initialization, yielding an -score of 0.670. While the fine-tuned multi-modal approaches led to the highest performance for CST regression, a significant improvement can be observed in the fine-tuned uni-modal approaches over their linear probing counterparts. We hypothesize that the features extracted by the encoders during linear probing may not fully capture the information needed for CST, which involves measuring the relative distance between the top and bottom of the retina-a task requiring more global context. This global context may not be adequately represented during contrastive pre-training. However, after fine-tuning, the model adjusts to integrate the necessary task-specific information, leading to improved performance.

Overall, the efficacy of various pre-training methods depends on factors like task difficulty and available sample size for training. Fully-supervised training or natural image-based pre-trained weights can yield results comparable to domain-specific pre-training under certain conditions. The absence of a definitive conclusion on the superior multi-modal pre-training method suggests that each exhibits unique strengths tailored to specific tasks. Nevertheless, our findings highlight the effectiveness of our approach and emphasize the significance of leveraging contrastive pre-training for enhancing model performance and feature extraction capabilities.

**Figure 5:**
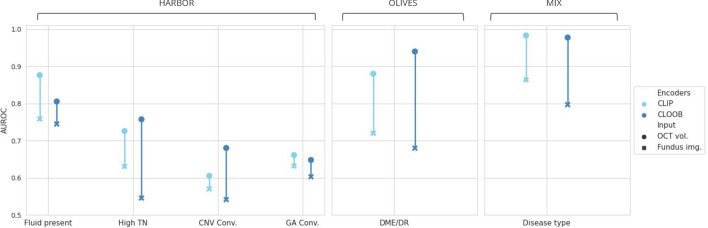
The predictive models’ performance on the external classification tasks when the fundus embeddings are used as classifier inputs.

### Interchanging retinal imaging modalities can preserve predictive power

The results of our investigation into the interchangeability of imaging modalities, particularly swapping OCT embeddings with fundus embeddings, are shown in Fig. 5. The performance decay on the different tasks is highly varied and especially large on the OLIVES dataset, which might be related to the quality of the acquired fundus images. Nevertheless, in most cases, this decay was $\le 20\%$, proving that interchangeability is somewhat possible. The performance drop is smaller in the case of CLIP-based pre-training, which was expected, as this pre-training approach yielded higher similarities between the corresponding fundus image and OCT volume embeddings. Although the fundus-image-based fine-tuned models achieve higher performance, by leveraging the acquired embeddings, we can bridge the gap between the two retinal imaging modalities, making it possible to extrapolate insights and predictions from one imaging modality to another. This versatility holds the potential to use predictive models in scenarios where access to the more expensive OCT imaging modality is limited or unavailable.

## Discussion

This work proposes a contrastive SSL pre-training method for ophthalmology. SSL holds significant promise for advancing the field, as multi-modal data, such as OCT volumes and fundus images, are often available, but annotations are scarce and costly. By leveraging large amounts of unlabeled data, we show that we can generate robust feature representations without the need for extensive manual labeling. Our pre-training applies multi-modal contrastive learning on a 3D OCT volume encoder and a 2D fundus image encoder, with the help of two contrastive objectives, InfoNCE and InfoLOOB, and in the case of the latter, the use of modern Hopfield layers to store reference embeddings.

Our method is designed to advance personalized patient management and disease progression modeling in ophthalmology by simultaneously considering different imaging modalities, namely OCT volumes and fundus images. Our overarching goal is to contribute meaningfully to the ongoing endeavors to establish multi-modal foundational models for various ophthalmological applications. It is important to note that our approach differs from the recent work conducted by Zhou et al.^34^, where they recently introduced a foundation model RETFound for 2D retinal images based on color fundus images and OCT B-scans; however, they built separate models for the two modalities, and as such, do not exploit the complementary information that these modalities can provide. Unlike their work, our study underscores the advantages of integrating volumetric data from OCT scans and effectively harmonizing information between these two essential modalities, paving the way for more comprehensive and robust medical imaging solutions.

The proposed self-supervised approach provides not only robust encoders to obtain comprehensive OCT and fundus image representations with interpretable features suitable for downstream clinical tasks but also a retrieval system. The research findings demonstrate that multi-modal contrastive pre-training enhances model performance across various clinically relevant supervised tasks, encompassing binary classification and regression. Our supervised models showcase remarkable robustness to challenges such as class imbalance and overfitting, even when dealing with limited data (treatment requirement, GA, and CNV prediction cases, for example). Additionally, the performance achieved by our models on external datasets is comparable to previous works, with some cases surpassing the reported results.

Our results align well with existing state-of-the-art methods across several studies. Kawczynski et al.^50^ used a ResNet50v2 CNN on 3D OCT volumes to predict BCVA scores, achieving $R^2 = 0.660$ (RMSE = 11.750), while our CLOOB-based model achieved $R^2 = 0.629$ (RMSE = 12.218). Romo-Bucheli et al.^46^ used a deep learning model on longitudinal OCT data for nAMD treatment prediction, with an AUROC of 0.810, compared to our CLOOB-based method at 0.755 and CLIP-based method at 0.796. Our model for predicting GA/CNV conversion over 24 months yielded AUROC scores of 0.701 and 0.698, paralleling Schmidt-Erfurt et al.^51^, but without extensive image segmentation. Finally, Kokilepersaud et al.^52^ used supervised contrastive learning on the OLIVES dataset, achieving an AUROC of around 0.800, which our multi-modal pre-trained models outperformed in DME/DR prediction tasks and matched in fundus image-based experiments.

In addition to the good performance across a diverse spectrum of clinically pertinent external downstream tasks, we conducted a feasibility analysis to explore the intriguing prospect of interchanging imaging modalities for predictive purposes. The fundamental attribute of contrastive learning, characterized by the close mapping of corresponding pairs within the latent space, empowers us to extend insights and predictions seamlessly from one imaging modality to another. Our results show a performance decay of $\le 20$%, underscoring the potential utility of predictive models even in clinical scenarios where the more resource-intensive OCT modality may be inaccessible. This emphasizes the robustness and adaptability of our approach in diverse clinical contexts.

Nonetheless, this work also has a few limitations. First, the contrastive pre-training of the encoders was done on data acquired from nAMD patients only, and it is unclear how training with a larger dataset encompassing various retinal diseases would affect the latent space. Second, we did not apply any mechanism to adjust for image domain shift resulting from using devices from several vendors for image acquisition, which was reflected in the latent space. This suggests that the models learn to extract relevant features from each type of image; however, they do not share much information between the vendors. Furthermore, while the subsampled OCT volumes used in this work capture more information about the retina’s structure and disease-related changes than existing 2D approaches that focus solely on the central B-scan, this may limit the model from reaching its full potential. Due to computational constraints, an often-faced disadvantage of SSL methods, we had to balance efficiency and accuracy by subsampling the OCT volumes. In future work, we plan to explore methods for encoding the full 3D OCT volume, which would enable the capture of more detailed spatial information and potentially enhance the model’s performance, particularly for tasks requiring comprehensive volumetric analysis. Lastly, we performed the training and evaluation of the supervised models in a cross-sectional manner, ignoring temporal correlations and treatment effects. Exploiting temporal modeling techniques could improve performance and should be further explored.

In conclusion, this work highlights the potential of multi-modal contrastive deep-learning models to leverage vast amounts of unlabeled data, offering promising avenues for various ophthalmology-image-interpretation tasks. This approach reduces the dependency on annotated datasets and mitigates inefficiencies in clinical workflows stemming from extensive labeling efforts. Our proposed method is a simple yet effective approach to developing 3D and multi-modal AI models and holds promise in advancing medical imaging tasks and facilitating more efficient and accurate patient care in ophthalmology. Future research should focus on enhancing the strength of multi-modal contrastive pre-training by addressing technical challenges related to limited training data and computational resources. Additionally, exploring the integration of further modalities to create robust and transferable encoders for predicting clinical visual function measures presents an intriguing avenue for further exploration in the clinical setting.

## Supplementary Information

Supplementary Information.

## Data Availability

The raw datasets are not publicly accessible and are the property of the respective companies. However, the data may be available from the Medical University of Vienna subject to local and national ethical approvals and can be requested from the authors via email at hrvoje.bogunovic@meduniwien.ac.at. The OLIVES dataset is publicly available at https://zenodo.org/records/7105232.

## Code availability

Implementing the CLIP/CLOOB-based contrastive pre-training was based on the code available on GitHub, accessible via this link https://github.com/ml-jku/cloob.
