# Supplementary material for: Multi-modal representation learning in retinal imaging using self-supervised learning for enhanced clinical predictions
Source: Sci Rep. 2024 Nov 5;14:26802. doi: 10.1038/s41598-024-78515-y (PMC11538269; doi:10.1038/s41598-024-78515-y)
Supplement: Supplementary file 1 — Supplementary Information. [file 41598_2024_78515_MOESM1_ESM.pdf]

Multi-Modal Representation Learning for Retinal Imaging: Leveraging Self-Supervised Learning for Enhanced Clinical Predictions

Emese Sükei[1,*], Elisabeth Rumetshofer[2], Niklas Schmidinger[2], Andreas Mayr[2], Ursula Schmidt-Erfurth[1], Günter Klambauer[2], and Hrvoje Bogunović[1,*]

[1]OPTIMA Lab, Department of Ophthalmology, Medical University of Vienna, Vienna, Austria
[2]LIT AI Lab, Institute for Machine Learning, Johannes Kepler University, Linz, Austria
[*]{emese.suekei, hrvoje.bogunovic}@meduniwien.ac.at

Supplementary figures

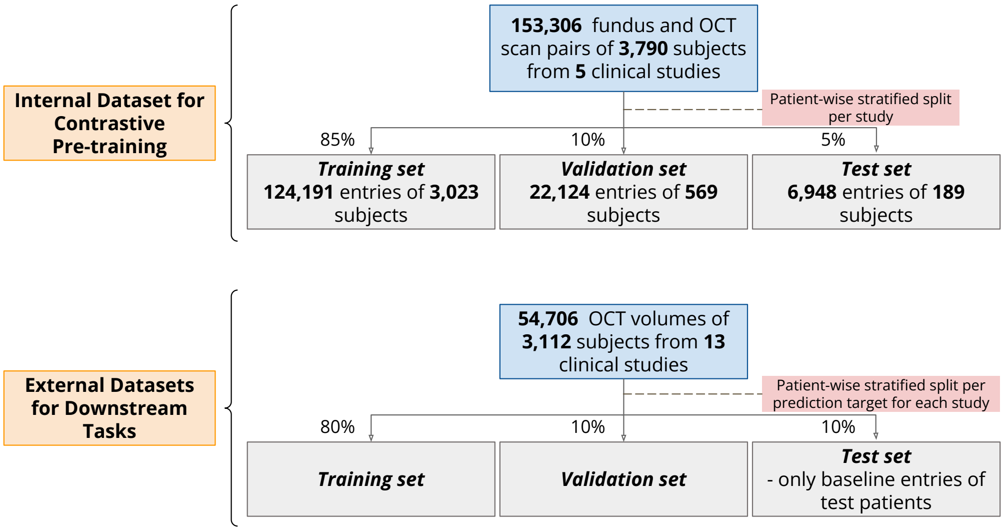

Figure A1. Flow chart of the data splits for the contrastive pre-training and external downstream task.

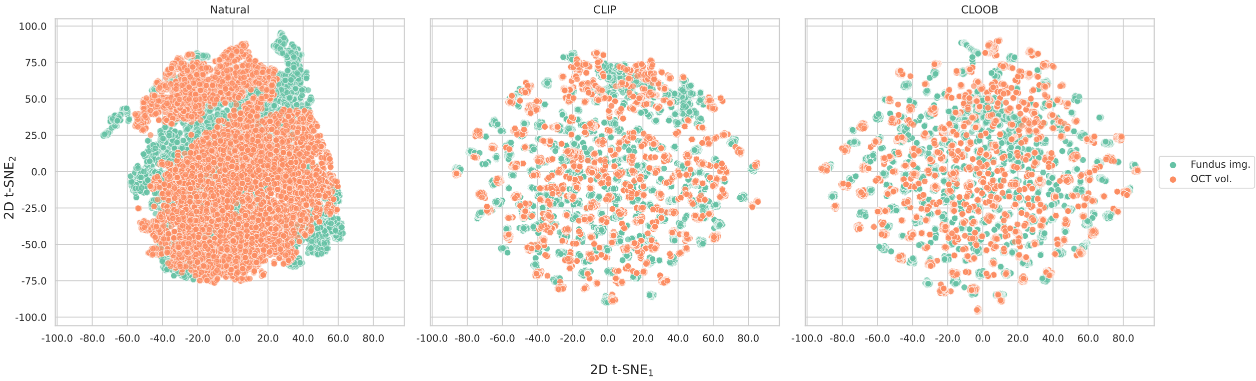

(a) t-SNE projections colored by imaging modality.

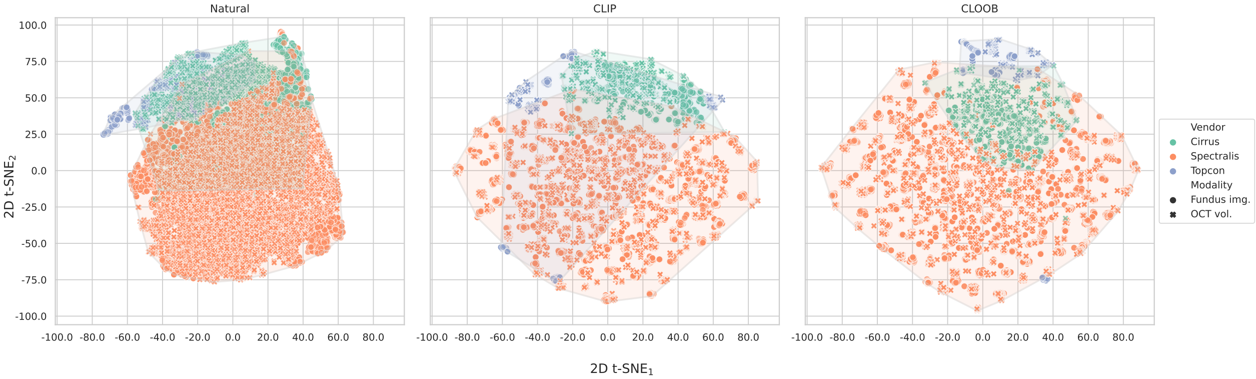

(b) t-SNE projections colored by imaging vendor.

Figure A2. Visualizations of latent representations using t-SNE projections created from the embeddings of two retinal imaging modalities in the hold-out test set. These visualizations compare embeddings obtained using natural image-based pre-trained weights with those obtained using `CLIP` and `CLOOB` contrastive weights for the 2D and 3D encoders.

Supplementary tables

Table A1. Overview of the datasets used in this study.

Variable	Pre-Training	HARBOR	OLIVES	MIX
**Overall dataset information**				
Patients, N	3,790	1,094	96	1,922
Eyes, N	7,023	2,183	96	1,922
- Study eye	3,789	1,094	96	1,495
- Fellow eye	3,234	1,089	0	427
Visits/patient, mean (SD)	16 (8)	24 (5)	17 (6)	1 (0)
Scans, N	153,306	51,186	1,590	1,929
- Study eye	113,940	25,676	1,590	1,502
- Fellow eye	39,366	25,510	0	427
Scanner used for imaging, N				
- Cirrus	21,776	51,186	0	976
- Spectralis	111,407	0	1,590	953
- Topcon	17,552	0	0	0
- Nidek	377	0	0	0
**Baseline demographic and clinical information**				
Gender, N				
- Male	0 (n/a)	444	38	186
- Female	0 (n/a)	650	32	241
- Unknown/not reported	3,790	0	26	1,495
Race, N				
- White	0 (n/a)	1,058	36	745
- Black or African American	0 (n/a)	5	22	68
- Asian	0 (n/a)	16	0 (n/a)	7
- Other	0 (n/a)	6	12	11
- Unknown/not reported	3,790	9	26	1,102
Age, mean (SD)	75 (8)	78 (8)	55 (11)	71 (11)
Disease, N				
- nAMD	3,790	1,094	0	0
- iAMD	0	0	0	418
- DR	0	0	40	0
- DME	0	0	56	757
- GA	0	0	0	170
- RVO	0	0	0	534
- Healthy	0	0	0	43

Table A2. Hyper-parameters used for the cross-modal contrastive pre-training.

	**Hyper-parameter**	**Value**
Learning	Optimizer	AdamW
	Learning rate	0.005
	Scheduler	Cosine annealing with restarts
	Weight decay	0.2
	Warm-up iterations	10000
Image encoder	Image resolution	224x224
	Model	ResNet18
Volume encoder	OCT volume resolution	224x224x20
	Model	VideoResNet18
Embedding space	Number of dimensions	512
InfoNCE	τ^{-1} - initial inverse temperature parameter (learnable)	14.3
InfoLOOB	τ^{-1} - inverse temperature parameter (fixed)	30
Hopfield layers	β - scaling parameter	14.3

Table A3. The mean performance of the predictive models with 95% confidence intervals on external downstream tasks using *linear probing*. These evaluations were conducted on the hold-out test set through bootstrapping-based validation. The highest performance for each training setup is highlighted in bold.

(a) Regression tasks

Dataset	Target	Encoder initialization	Model input			
			Fundus (2D)		OCT (3D)	
			RMSE	R^2	RMSE	R^2
HARBOR	BCVA	`Natural`	20.167 (18.395, 21.843)	-0.011 (-0.126, 0.082)	16.848 (15.469, 18.294)	0.294 (0.203, 0.373)
		`SimCLR`	20.240 (18.489, 21.893)	-0.018 (-0.139, 0.079)	16.776 (15.344, 18.176)	0.300 (0.195, 0.390)
		`BYOL`	20.295 (18.558, 21.886)	-0.024 (-0.123, 0.062)	19.091 (17.394, 20.671)	0.094 (0.003, 0.171)
		`VICReg`	20.552 (18.731, 22.233)	-0.050 (-0.163, 0.046)	17.026 (15.549, 18.502)	0.279 (0.186, 0.360)
		`CLIP`	**16.688 (15.252, 18.110)**	**0.308 (0.171, 0.415)**	**14.715 (13.415, 15.957)**	**0.462 (0.362, 0.545)**
		`CLOOB`	16.914 (15.506, 18.275)	0.289 (0.166, 0.394)	16.814 (15.504, 18.118)	0.297 (0.144, 0.417)
	CST [µm]	`Natural`	129.552 (115.325, 143.722)	-0.428 (-0.554, -0.320)	107.613 (96.384, 118.832)	0.015 (-0.083, 0.107)
		`SimCLR`	128.594 (114.808, 142.596)	-0.407 (-0.531, -0.298)	108.687 (97.101, 120.399)	-0.005 (-0.117, 0.096)
		`BYOL`	127.265 (112.782, 141.794)	-0.378 (-0.503, -0.270)	127.743 (113.303, 142.212)	-0.388 (-0.497, -0.293)
		`VICReg`	123.926 (109.984, 137.848)	-0.306 (-0.420, -0.207)	113.371 (101.325, 125.538)	-0.094 (-0.211, 0.010)
		`CLIP`	120.337 (107.494, 133.509)	-0.232 (-0.351, -0.135)	91.724 (81.937, 101.910)	0.284 (0.208, 0.351)
		`CLOOB`	**116.597 (104.048, 129.646)**	**-0.157 (-0.283, -0.055)**	**81.800 (73.921, 89.474)**	**0.431 (0.348, 0.497)**
OLIVES	BCVA	`Natural`	15.238 (11.127, 18.631)	-1.360 (-4.330, -0.236)	8.016 (5.783, 9.925)	0.347 (-0.386, 0.579)
		`SimCLR`	11.383 (6.562, 15.500)	-0.317 (-1.788, 0.420)	9.042 (5.884, 11.640)	0.169 (-0.266, 0.373)
		`BYOL`	**9.804 (5.612, 13.449)**	**0.023 (-0.865, 0.471)**	8.178 (5.717, 10.335)	0.320 (-0.020, 0.438)
		`VICReg`	10.648 (6.851, 13.993)	-0.152 (-1.293, 0.366)	8.182 (4.886, 11.197)	0.320 (-0.244, 0.587)
		`CLIP`	13.010 (9.179, 16.500)	-0.720 (-3.320, 0.159)	9.956 (7.062, 12.657)	-0.007 (-2.117, 0.605)
		`CLOOB`	11.116 (6.685, 14.904)	-0.256 (-1.727, 0.439)	**7.910 (6.052, 9.516)**	**0.364 (-0.532, 0.628)**
	CST [µm]	`Natural`	152.908 (107.051, 193.098)	-0.095 (-0.866, 0.276)	128.607 (78.947, 172.238)	0.226 (-0.305, 0.526)
		`SimCLR`	202.449 (127.550, 264.305)	-0.919 (-2.118, -0.078)	132.526 (84.129, 173.050)	0.178 (-0.248, 0.444)
		`BYOL`	169.763 (110.201, 220.100)	-0.349 (-1.152, 0.061)	130.553 (73.208, 175.565)	0.202 (-0.328, 0.558)
		`VICReg`	158.861 (104.134, 204.929)	-0.182 (-0.851, 0.118)	131.744 (84.352, 171.721)	0.187 (-0.355, 0.478)
		`CLIP`	**123.492 (78.700, 160.497)**	**0.286 (-0.200, 0.598)**	80.782 (54.066, 103.429)	0.694 (0.553, 0.787)
		`CLOOB`	150.215 (105.563, 187.405)	-0.056 (-0.895, 0.322)	**53.995 (35.282, 73.430)**	**0.864 (0.748, 0.930)**

(b) Classification and forecasting tasks

Dataset	Target	Encoder initialization	Model input			
			Fundus (2D)		OCT (3D)	
			AUROC	AP	AUROC	AP
HARBOR	Fluid present	`Natural`	0.574 (0.503, 0.646)	0.873 (0.832, 0.912)	0.713 (0.652, 0.775)	0.921 (0.891, 0.948)
		`SimCLR`	0.545 (0.460, 0.623)	0.842 (0.789, 0.890)	0.782 (0.724, 0.832)	0.942 (0.917, 0.963)
		`BYOL`	0.588 (0.512, 0.665)	0.862 (0.814, 0.905)	0.574 (0.492, 0.648)	0.834 (0.778, 0.889)
		`VICReg`	0.544 (0.460, 0.622)	0.844 (0.794, 0.893)	0.717 (0.654, 0.783)	0.920 (0.889, 0.948)
		`CLIP`	0.775 (0.722, 0.827)	0.944 (0.924, 0.963)	**0.871 (0.833, 0.907)**	**0.971 (0.959, 0.981)**
		`CLOOB`	**0.776 (0.715, 0.833)**	**0.939 (0.913, 0.962)**	0.804 (0.745, 0.854)	0.943 (0.913, 0.967)
	High TR	`Natural`	0.528 (0.409, 0.650)	0.209 (0.118, 0.374)	0.596 (0.469, 0.726)	0.292 (0.178, 0.484)
		`SimCLR`	0.565 (0.453, 0.676)	0.185 (0.121, 0.309)	0.550 (0.412, 0.680)	0.216 (0.125, 0.385)
		`BYOL`	0.597 (0.457, 0.734)	0.234 (0.139, 0.410)	0.591 (0.466, 0.719)	0.221 (0.137, 0.379)
		`VICReg`	0.487 (0.352, 0.623)	0.170 (0.103, 0.294)	0.591 (0.464, 0.711)	0.224 (0.135, 0.393)
		`CLIP`	**0.633 (0.507, 0.755)**	**0.259 (0.154, 0.441)**	0.709 (0.564, 0.832)	0.429 (0.253, 0.647)
		`CLOOB`	0.622 (0.502, 0.743)	0.285 (0.177, 0.469)	**0.755 (0.652, 0.846)**	**0.323 (0.205, 0.512)**
	GA Conv.	`Natural`	0.539 (0.392, 0.690)	0.238 (0.115, 0.444)	0.467 (0.342, 0.600)	0.136 (0.081, 0.282)
		`SimCLR`	0.448 (0.317, 0.591)	0.138 (0.075, 0.282)	0.607 (0.482, 0.717)	0.164 (0.098, 0.301)
		`BYOL`	0.510 (0.373, 0.646)	0.138 (0.084, 0.250)	0.468 (0.335, 0.604)	0.117 (0.072, 0.207)
		`VICReg`	0.589 (0.448, 0.720)	0.175 (0.106, 0.308)	0.524 (0.397, 0.647)	0.132 (0.083, 0.242)
		`CLIP`	0.537 (0.408, 0.660)	0.182 (0.091, 0.326)	**0.665 (0.533, 0.795)**	**0.238 (0.132, 0.432)**
		`CLOOB`	**0.555 (0.431, 0.684)**	**0.207 (0.100, 0.377)**	0.648 (0.509, 0.773)	0.200 (0.119, 0.369)
	CNV Conv.	`Natural`	0.524 (0.422, 0.621)	0.174 (0.119, 0.270)	0.591 (0.474, 0.702)	0.233 (0.145, 0.385)
		`SimCLR`	0.565 (0.463, 0.666)	0.195 (0.133, 0.307)	0.513 (0.413, 0.614)	0.177 (0.118, 0.287)
		`BYOL`	0.528 (0.421, 0.640)	0.191 (0.127, 0.312)	0.522 (0.405, 0.634)	0.192 (0.128, 0.306)
		`VICReg`	0.545 (0.432, 0.659)	0.200 (0.130, 0.319)	0.461 (0.360, 0.561)	0.156 (0.109, 0.238)
		`CLIP`	**0.581 (0.462, 0.696)**	**0.279 (0.165, 0.439)**	0.605 (0.495, 0.714)	0.239 (0.158, 0.392)
		`CLOOB`	0.560 (0.438, 0.679)	0.262 (0.154, 0.424)	**0.674 (0.585, 0.764)**	**0.247 (0.168, 0.379)**
OLIVES	DME/DR	`Natural`	0.700 (0.389, 0.944)	0.519 (0.226, 0.929)	0.640 (0.166,1.0)	0.689 (0.187, 1.00)
		`SimCLR`	0.700 (0.320, 1.0)	0.655 (0.236, 1.0)	0.600 (0.273, 0.907)	0.446 (0.194, 0.886)
		`BYOL`	0.860 (0.593, 1.0)	0.789 (0.375, 1.0)	0.820 (0.538, 1.0)	0.739 (0.298, 1.0)
		`VICReg`	0.740 (0.417, 1.0)	0.572 (0.250, 1.0)	0.620 (0.280, 0.923)	0.463 (0.200, 0.900)
		`CLIP`	0.740 (0.428, 0.962)	0.544 (0.250, 0.952)	0.880 (0.667, 1.0)	0.768 (0.392, 1.0)
		`CLOOB`	**0.940 (0.769, 1.0)**	**0.925 (0.667, 1.0)**	**0.940 (0.769, 1.0)**	**0.877 (0.533, 1.0)**
MIX	Disease type	`Natural`	0.875 (0.853, 0.895)	0.667 (0.610, 0.716)	0.928 (0.912, 0.941)	0.773 (0.726, 0.816)
		`SimCLR`	0.617 (0.581, 0.654)	0.270 (0.237, 0.311)	0.946 (0.934, 0.957)	0.837 (0.800, 0.871)
		`BYOL`	0.863 (0.841, 0.882)	0.606 (0.548, 0.660)	0.861 (0.839, 0.881)	0.598 (0.541, 0.654)
		`VICReg`	0.868 (0.847, 0.886)	0.619 (0.558, 0.672)	0.925 (0.908, 0.940)	0.787 (0.740, 0.824)
		`CLIP`	**0.929 (0.913, 0.942)**	0.789 (0.744, 0.828)	**0.982 (0.977, 0.987)**	**0.940 (0.921, 0.957)**
		`CLOOB`	0.928 (0.913, 0.943)	**0.791 (0.745, 0.830)**	0.978 (0.971, 0.983)	0.924 (0.901, 0.944)

Table A4. The mean performance of the predictive models with 95% confidence intervals on external downstream tasks using *fine-tuning*. These evaluations were conducted on the hold-out test set through bootstrapping-based validation. The highest performance for each training setup is highlighted in bold.

(a) Regression tasks

Dataset	Target	Encoder initialization	Model input			
			Fundus (2D)		OCT (3D)	
			RMSE	R^2	RMSE	R^2
HARBOR	BCVA	Random	21.765 (20.071,23.347)	-0.177 (-0.348, -0.033)	12.098 (10.727, 13.445)	0.636 (0.547, 0.709)
		Natural	17.221 (15.684, 18.587)	0.263 (0.133, 0.374)	11.689 (10.538, 12.751)	0.660 (0.584,0.721)
		SimCLR	16.628 (15.101, 18.046)	0.313 (0.183, 0.423)	**11.512 (10.142, 12.804)**	**0.670 (0.585, 0.740)**
		BYOL	17.355 (15.770, 18.897)	0.251 (0.112, 0.369)	12.408 (11.185, 13.604)	0.617 (0.539, 0.685)
		VICReg	17.337 (15.807, 18.839)	0.253 (0.109, 0.365)	13.307 (11.865, 14.611)	0.560 (0.457, 0.651)
		CLIP	**16.310 (14.949, 17.697)**	**0.339 (0.208, 0.449)**	12.323 (11.108, 13.442)	0.622 (0.545, 0.691)
		CLOOB	16.423 (14.973, 17.834)	0.329 (0.192, 0.443)	12.218 (11.006, 13.377)	0.629 (0.539, 0.699)
	CST [µm]	Random	161.234 (147.385, 175.923)	-1.211 (-1.589,-0.911)	85.020 (76.070, 93.343)	0.384 (0.292,0.464)
		Natural	125.638 (112.945, 138.954)	-0.343 (-0.499, -0.209)	74.160 (66.118, 81.705)	0.532 (0.430, 0.612)
		SimCLR	131.279 (116.946, 145.467)	-0.466 (-0.613, -0.344)	78.870 (70.879, 86.749)	0.470 (0.378, 0.550)
		BYOL	129.915 (115.821, 144.250)	-0.436 (-0.576, -0.315)	80.323 (59.472,107.030)	0.451 (-0.013,0.702)
		VICReg	129.567 (116.441, 142.717)	-0.428 (-0.596,-0.277)	73.131 (64.730, 81.672)	0.545 (0.447, 0.622)
		CLIP	**112.938 (100.536, 125.803)**	**-0.085 (-0.176, -0.005)**	64.870 (52.061, 81.038)	0.642 (0.422, 0.777)
		CLOOB	122.770 (108.843, 136.728)	-0.282 (-0.385, -0.195)	**64.298 (51.620, 80.412)**	**0.648 (0.427, 0.776)**
OLIVES	BCVA	Random	14.985 (8.814, 21.010)	-1.282 (-4.214,0.072)	7.224 (4.479, 9.480)	0.470 (0.056, 0.673)
		Natural	9.858 (6.901, 12.582)	0.012 (-1.078, 0.429)	6.851 (3.700, 9.493)	0.523 (0.018, 0.802)
		SimCLR	9.236 (5.761, 12.083)	0.133 (-0.416, 0.418)	7.159 (4.596, 9.728)	0.479 (-0.154, 0.746)
		BYOL	10.851 (6.946, 14.309)	-0.197 (-1.224, 0.298)	8.551 (5.333, 11.713)	0.257 (-0.661, 0.676)
		VICReg	9.145 (6.325, 11.624)	0.150 (-0.740, 0.480)	7.111 (4.146, 10.087)	0.486 (-0.185, 0.783)
		CLIP	9.347 (5.911, 12.387)	0.112 (-0.574, 0.412)	5.831 (3.000, 8.051)	0.654 (0.029, 0.909)
		CLOOB	**7.454 (5.250, 9.500)**	**0.435 (-0.027, 0.622)**	**5.601 (3.373, 7.467)**	**0.681 (0.306, 0.856)**
	CST [µm]	Random	238.450 (166.010, 299.520)	-1.662 (-3.328, -0.982)	86.648 (50.785, 118.532)	0.648 (0.389, 0.808)
		Natural	135.106 (94.991, 170.290)	0.145 (-0.376, 0.385)	77.561 (45.622, 104.604)	0.718 (0.514, 0.854)
		SimCLR	161.251 (103.507, 208.809)	-0.217 (-1.009, 0.186)	39.625 (24.143, 53.056)	0.926 (0.823, 0.972)
		BYOL	175.295 (111.847, 227.131)	-0.439 (-1.317, -0.023)	61.765 (37.794, 81.894)	0.821 (0.701, 0.902)
		VICReg	174.455 (104.595, 232.826)	-0.425 (-1.367, 0.113)	51.114 (33.464, 66.857)	0.878 (0.778, 0.935)
		CLIP	122.149 (76.378, 160.604)	0.301 (-0.150, 0.553)	29.099 (22.088, 34.847)	0.960 (0.924, 0.976)
		CLOOB	**110.547 (75.461, 139.629)**	**0.428 (0.091, 0.590)**	**28.106 (19.962, 35.386)**	**0.963 (0.916, 0.982)**

(b) Classification and forecasting tasks

Dataset	Target	Encoder initialization	Model input			
			Fundus (2D)		OCT (3D)	
			AUROC	AP	AUROC	AP
HARBOR	Fluid present	Random	0.580 (0.507, 0.652)	0.871 (0.827, 0.912)	0.906 (0.870, 0.936)	0.977 (0.966, 0.987)
		Natural	0.720 (0.646, 0.791)	0.890 (0.846, 0.939)	0.900 (0.869, 0.931)	0.978 (0.969,0.986)
		SimCLR	0.667 (0.600, 0.736)	0.903 (0.869, 0.935)	0.918 (0.890, 0.944)	0.982 (0.974, 0.989)
		BYOL	0.709 (0.639, 0.775)	0.909 (0.870, 0.943)	0.910 (0.880, 0.938)	0.980 (0.971, 0.987)
		VICReg	0.616 (0.542, 0.693)	0.867 (0.819, 0.914)	0.896 (0.863, 0.926)	0.977 (0.967, 0.985)
		CLIP	**0.805 (0.753, 0.856)**	**0.951 (0.931, 0.969)**	**0.930 (0.903, 0.953)**	**0.985 (0.978, 0.991)**
		CLOOB	0.775 (0.720, 0.829)	0.941 (0.919, 0.962)	0.915 (0.886, 0.940)	0.982 (0.974, 0.989)
	High TR	Random	0.529 (0.389, 0.672)	0.227 (0.130, 0.421)	0.546 (0.430, 0.668)	0.223 (0.141, 0.391)
		Natural	0.542 (0.412, 0.669)	0.191 (0.117, 0.336)	0.526 (0.394, 0.645)	0.175 (0.112, 0.297)
		SimCLR	0.293 (0.183, 0.411)	0.116 (0.076, 0.187)	0.626 (0.498, 0.746)	0.265 (0.168, 0.427)
		BYOL	0.441 (0.312, 0.586)	0.151 (0.098, 0.237)	0.583 (0.457, 0.700)	0.237 (0.149, 0.399)
		VICReg	0.503 (0.373, 0.632)	0.174 (0.111, 0.309)	0.509 (0.368, 0.654)	0.222 (0.127, 0.398)
		CLIP	**0.660 (0.527, 0.783)**	**0.344 (0.191, 0.534)**	**0.796 (0.683, 0.887)**	**0.435 (0.282, 0.664)**
		CLOOB	0.553 (0.419, 0.699)	0.231 (0.139, 0.422)	0.762 (0.659, 0.856)	0.398 (0.236, 0.597)
	GA Conv.	Random	0.619 (0.497, 0.736)	0.173 (0.103, 0.313)	0.565 (0.424, 0.701)	0.161 (0.094, 0.307)
		Natural	0.602 (0.430, 0.758)	0.237 (0.121, 0.447)	0.568 (0.447, 0.695)	0.171 (0.099, 0.329)
		SimCLR	0.566 (0.443, 0.693)	0.149 (0.090, 0.272)	0.568 (0.459, 0.685)	0.160 (0.096, 0.308)
		BYOL	0.565 (0.426, 0.706)	0.171 (0.099, 0.333)	0.527 (0.394, 0.660)	0.135 (0.082, 0.243)
		VICReg	0.563 (0.428, 0.693)	0.157 (0.092, 0.290)	0.519 (0.376, 0.672)	0.148 (0.090, 0.271)
		CLIP	**0.636 (0.522, 0.745)**	**0.182 (0.106, 0.321)**	**0.701 (0.593, 0.802)**	**0.224 (0.135, 0.404)**
		CLOOB	0.632 (0.494, 0.752)	0.199 (0.110, 0.367)	0.693 (0.586, 0.797)	0.210 (0.130, 0.369)
	CNV Conv.	Random	0.526 (0.419, 0.630)	0.179 (0.122, 0.276)	0.614 (0.506, 0.721)	0.266 (0.158, 0.420)
		Natural	0.492 (0.387, 0.596)	0.175 (0.116, 0.288)	0.626 (0.524, 0.725)	0.239 (0.159, 0.384)
		SimCLR	0.419 (0.304, 0.536)	0.160 (0.105, 0.264)	0.565 (0.451, 0.678)	0.213 (0.137, 0.349)
		BYOL	0.534 (0.425, 0.644)	0.192 (0.130, 0.309)	0.540 (0.430, 0.654)	0.293 (0.166, 0.436)
		VICReg	0.478 (0.362, 0.603)	0.184 (0.121, 0.304)	0.559 (0.440, 0.676)	0.206 (0.133, 0.325)
		CLIP	0.575 (0.470, 0.683)	0.200 (0.135, 0.306)	0.647 (0.541, 0.751)	0.280 (0.175, 0.429)
		CLOOB	**0.621 (0.521, 0.725)**	**0.236 (0.157, 0.377)**	**0.698 (0.595, 0.793)**	**0.299 (0.202, 0.463)**
OLIVES	DME/DR	Random	0.640 (0.240, 1.0)	0.627 (0.201, 1.0)	0.920 (0.714, 1.0)	0.885 (0.583, 1.0)
		Natural	0.680 (0.340, 0.955)	0.578 (0.208, 0.957)	0.900 (0.630, 1.0)	0.900 (0.591, 1.0)
		SimCLR	0.620 (0.231, 0.944)	0.570 (0.188, 0.933)	0.920 (0.727, 1.0)	0.876 (0.500, 1.0)
		BYOL	0.360 (0.077, 0.680)	0.306 (0.133, 0.649)	0.880 (0.636, 1.0)	0.796 (0.352, 1.0)
		VICReg	0.360 (0.091, 0.679)	0.302 (0.140, 0.617)	0.720 (0.432, 0.964)	0.505 (0.242, 0.964)
		CLIP	0.840 (0.577, 1.0)	0.743 (0.361, 1.0)	0.980 (0.889, 1.0)	0.967 (0.833, 1.0)
		CLOOB	**0.960 (0.833, 1.0)**	**0.943 (0.733, 1.0)**	**1.0 (1.0, 1.0)**	**1.0 (0.999, 1.0)**
MIX	Disease type	Random	0.881 (0.861, 0.899)	0.655 (0.600, 0.709)	0.966 (0.956, 0.975)	0.899 (0.872, 0.924)
		Natural	0.925 (0.907, 0.941)	0.796 (0.754, 0.835)	0.986 (0.980, 0.990)	0.954 (0.937, 0.968)
		SimCLR	0.918 (0.899, 0.932)	0.747 (0.695, 0.793)	0.987 (0.981, 0.992)	0.958 (0.942, 0.973)
		BYOL	0.890 (0.870, 0.909)	0.675 (0.617, 0.727)	0.991 (0.987, 0.994)	0.969 (0.957, 0.980)
		VICReg	0.892 (0.873, 0.909)	0.691 (0.637, 0.737)	0.989 (0.983, 0.993)	0.964 (0.949, 0.976)
		CLIP	0.950 (0.937, 0.960)	0.845 (0.807, 0.878)	0.993 (0.990, 0.995)	0.973 (0.962, 0.982)
		CLOOB	**0.960 (0.949, 0.969)**	**0.878 (0.845, 0.905)**	**0.995 (0.993, 0.997)**	**0.982 (0.973, 0.989)**
